# Proteomic analysis of lung cancer cells reveals a critical role of BCAT1 in cancer cell metastasis

**DOI:** 10.7150/thno.61731

**Published:** 2021-09-27

**Authors:** Lin Mao, Jin Chen, Xue Lu, Canlin Yang, Yi Ding, Mingming Wang, Yunpeng Zhang, Yuying Tian, Xing Li, Yunyun Fu, Yueying Yang, Yunyun Gu, Fei Gao, Junxing Huang, Lujian Liao

**Affiliations:** 1 Shanghai Key Laboratory of Regulatory Biology, School of Life Sciences, East China Normal University, Shanghai 200241, China.; 2 Department of Oncology, Taizhou People's Hospital, Affiliated to Nanjing University of Chinese Medicine, Taizhou, Jiangsu 225300, China.; 3 Durbrain Medical Laboratory, Hangzhou, Zhejiang 310000, China.

**Keywords:** BCAT1, SOX2, stemness, metastasis

## Abstract

Metastasis is the major cause of high mortality in lung cancer. Exploring the underlying mechanisms of metastasis thus holds promise for identifying new therapeutic strategies that may enhance survival.

**Methods:** We applied quantitative mass spectrometry to compare protein expression profiles between primary and metastatic lung cancer cells whilst investigating metastasis-related molecular features.

**Results:** We discovered that BCAT1, the key enzyme in branched-chain amino acid metabolism, is overexpressed at the protein level in metastatic lung cancer cells, as well as in metastatic tissues from lung cancer patients. Analysis of transcriptomic data available in the TCGA database revealed that increased BCAT1 transcription is associated with poor overall survival of lung cancer patients. In accord with a critical role in metastasis, shRNA-mediated knockdown of BCAT1 expression reduced migration of metastatic cells *in vitro* and the metastasis of these cells to distal organs in nude mice. Mechanistically, high levels of BCAT1 depleted α-ketoglutarate (α-KG) and promoted expression of SOX2, a transcription factor regulating cancer cell stemness and metastasis.

**Conclusion:** Our findings suggest that BCAT1 plays an important role in promoting lung cancer cell metastasis, and may define a novel pathway to target as an anti-metastatic therapy.

## Introduction

Lung cancer is one of the leading causes of cancer-related death 1 and metastasis of lung cancer cells from primary tumors in the lung to distal organs is the major driver of its high mortality rate. Diagnosis of lung cancer is disproportionately made at late-stages of the disease, rendering surgical treatment unfeasible. Late diagnosis and the critical role of metastasis in patient survival create a great and, as of yet, unmet need for therapies that can prevent lung cancer metastasis. Understanding the molecular profiles of metastatic tumors is the first step to identify key pathways that might be exploited for this purpose. Over the past decade, a number of studies have applied genome sequencing to understand the mutational profiles of metastatic tumors 2, 3. Recently, comprehensive proteogenomic studies on lung cancer patient specimens have rendered the proteome landscape of primary tumors relative to para-tumor “normal” tissues 4, 5. However, similar studies at the proteome level on metastatic lung cancers are still missing.

Amino acid metabolism plays critical roles in tumorigenesis and metastasis. Among them, the transamination reaction from branched-chain amino acids (BCAA) to α-ketoglutarate (α-KG) is catalyzed by either of two types of BCATs, the cytosolic BCAT1 and mitochondrial BCAT2. This reaction produces glutamate, which is synthesized from multiple reactions and participates in important metabolic pathways preferentially utilized by tumor cells to promote survival 6. The resulting branched-chain keto acids (BCKA) are further catabolized to acetyl- and succinyl-CoA which are intermediates in the TCA cycle 7. Overexpression of BCAT1 has been associated with cancer progression in myeloid leukemia 8, glioma 9 and non-small cell lung cancer (NSCLC) 10, and increased uptake of BCAA is important for maintaining tumorigenesis in NSCLC 11. However, whether BCAT1 expression is dysregulated in metastatic tumors and plays a role in underlying processes of migration, remains unclear.

In this study, we applied quantitative mass spectrometry to compare metastatic lung cancer cells to primary lung cancer cells, and bioinformatic analyses were performed to delineate the unique molecular features of metastatic cells. From the pool of proteins that are significantly changed in metastatic cells, we found that overexpression of BCAT1 was closely associated with metastatic state. Utilizing *in vitro* assays of cell migration, we further demonstrated that increased cancer cell progression is mechanistically linked to BCAT1-mediated regulation of SOX2 expression. Thus, our study provides a comprehensive look at proteome changes associated with the metastatic state in lung cancer, and define a novel role of amino acid metabolism that may offer new therapeutic targets to mitigate metastasis.

## Materials and Methods

### Animals

All animal experiments were performed in accordance with animal protocol approved by the Ethics Committee of East China Normal University. 4~6 week-old Female BALB/c Nude mice were purchased from Sino-British Sippr/BK Lab Animal Co, Ltd (Shanghai, China). Animals were housed in standard mouse cage under conditions of optimum light, temperature, and humidity, with plenty of food and water.

### Ethics on human samples

Four pairs of formalin-fixed, paraffin-embedded (FFPE) tumor sections were procured, with each pair of primary and metastatic tissue coming from the same patient. An additional pair of frozen tumor tissues consisted of one primary and one lymph node-metastatic tumor from separate patients. Together the 10 tissues were used for tandem mass tag (TMT)-based quantitative proteomic analysis. FFPE samples were obtained from the Department of Oncology, Taizhou People's Hospital and the frozen samples were from Shanghai Chest Hospital. The study was approved by the institutional review committees of both hospitals.

### Stable isotope labeling of amino acids in cell culture (SILAC) and proteomic analysis

The NSCLC cell line A549 (L0) and derived metastasis clones (L2, L6) were obtained from Dr. Luo Jian (East China Normal University, China). A549 L2 cells were metabolically labeled with “medium” heavy ^13^C_6_-arginine and D_4_-lysine, while L6 cells were labeled with heavy ^13^C_6_^15^N_4_-arginine and ^13^C_6_^15^N_2_-lysine (Cambridge Isotope Laboratories, USA). L0 cells were cultured with “light” amino acids. All cells were cultured in DMEM with 10% dialyzed fetal bovine serum and 1% penicillin and streptomycin at 37 °C in 5% CO_2_. Harvesting of cells for LC-MS/MS followed the same procedure as described previously 12.

### TMT based proteomics analysis of tumor tissues

FFPE tissues were dewaxed by heptane with vortexing for 10 min, and centrifuged at 10000 × g for 2 min. The supernatant was removed and the process was repeated once. The tissues were incubated in dewaxing buffer overnight. Both FFPE and frozen tissues were then suspended in lysis buffer (4% SDS, 100 mM Tris-HCl, pH 8.0), homogenized for 2 min with 70 Hz (Cat. #JXFSTPRP-24/32, Jingxin, China) and incubated at 95 °C for 60 min, followed by sonication on ice for 2 min and centrifugation at 16000 × g for 10 min at 4 °C. The supernatant protein concentration was quantified using BCA (Cat. #23225, Thermo Fisher Scientific, USA), then 250 μg of protein was reduced with 100 mM of DTT in 95 °C for 10 min. Equal volumes of urea buffer (8 M urea, 100 mM Tris-HCl pH 8.5) were added and the samples were transferred to 30-kDa micron filters (MRCF0R030, Millipore, USA), followed by centrifugation at 14000 × g for 15 min. Proteins were alkylated with 50 mM iodoacetamide in urea buffer for 20 min, and the buffer was exchanged to 0.1 M TEAB (Cat. #140023, Sigma-Aldrich, USA) by centrifugation at 14000 × g for 15 min with 4 repetitions. Samples were digested overnight at 37 °C with trypsin, desalted and labeled by a 10-plex tandem mass tag (TMT) labeling reagent (Cat. #90110, Thermo Scientific, USA) following the manufacturer's instructions. The remaining steps of offline fractionation and LC-MS/MS analysis were essentially the same as previously described 13.

### MS data analysis

The MaxQuant (version 1.4.1.2) software was used for proteomic data analysis. MS/MS spectra were searched against a UniportKB/Swiss-Prot human database. The precursor mass tolerance was set to 15 ppm, and trypsin was set as the protease. Two missed cleavages were allowed. The false discovery rate (FDR) was set to 1% at both the peptide and protein level. For SILAC quantification, multiplicity was set to triple SILAC labelling (Lys+0/Arg+0, Lys+4/Arg+6, Lys+8/Arg+10). Oxidation (+15.9949 Da) of methionine, and carbamidomethylation (+57.0215 Da) of cysteine were set as static modifications. For TMT data, TMT 10-plex (+229.1629 Da) on lysine and N-terminus were added as static modifications.

### Statistical analysis

Data analysis was performed using R version 4.0.3 (R-Core-Team, 2020). Proteins with missing values were excluded, differential protein expression was determined using *t*-test. Principal component analysis (PCA) was conducted to demonstrate overall differences of samples, and enrichment analysis was performed with the package clusterProfiler 14 and David bioinformatics resource v6.8 (https://david.ncifcrf.gov/). Network analysis was conducted in STRING (https://string-db.org), and exported using Cytoscape v3.8.2.

For Kaplan-Meier survival analysis, samples were stratified according to BCAT1 expression: samples with BCAT1 expression above the 75% quantile were considered as the high-expression cohort, while samples with BCAT1 expression lower than 25% were defined as the low-expression cohort. Differences in survival time were analyzed by the log-rank test, and the Cox proportional hazard model was used to evaluate the impact of BCAT1 expression on survival time. A 95% confidence interval was drawn around the survival curves. The analysis was conducted using R (4.0.3) from the website GEPIA (http://gepia.cancer-pku.cn/). Data was from TCGA and published datasets Cell, 2020 4.

Quantative results were analysed by two-tailed unpaired or paired (in case of paired samples) student's *t*-test using GraphPad Prism 8.0.

### Bioluminescence imaging

A549 cells stably expressing luciferase as well as either plKO.1-shSCR (scramble), plKO.1-shBCAT1#1, or plKO.1-shBCAT1#2 were delivered into mice. Each mouse received a total of 1 × 10^5^ cells suspended in 100 μL PBS via injection into the left ventricle. Four weeks after injection, metastasis was monitored by bioluminescent imaging (Caliper Life Sciences, Hopkinton, MA), after intraperitoneal injection of D-luciferin potassium (AOK Chem, China) into the mice at a dose of 150 mg/kg. Bioluminescence images (BLI) were acquired and quantified using Living Image 3.2 (Caliper Life Sciences, Hopkinton, MA); images were captured using automatic exposure settings. The intensities within fixed regions of interest (ROI) fluorescence signals were measured. Normalization of the BLI signal from each mouse was achieved by dividing BLI intensity by that acquired from the same mouse on day 0 after injection. The mice were then sacrificed, and organs were dissected for further analysis of metastasis.

### Constructs and lentiviral production

Short hairpin RNA constructs against BCAT1 (shBCAT1) were cloned in plKO.1 vector using AgeI and EcoRI cloning sites. Oligonucleotide sequences used to target BCAT1 are listed in Table S1. Lentiviral particles were produced by co-transfection of HEK293t with the packaging plasmid psPAX2, the envelope plasmid pMD2.G, and the respective plKO.1shRNA containing a BCAT1-targeting sequence or scrambled sequence. Virus was collected 2 days after transfection. A549 cells were treated with the virus and stably infected single clones were selected by 1 μg/mL puromycin (Cat. #60210ES25, YEASEN, China).

### Quantitation of cellular organic acids

Cells (~ 2 × 10^6^ cells per sample) were harvested and lysed in buffer (H_2_O:MeOH:ACN = 1:2:2), frozen in liquid nitrogen, then thawed and sonicated at 4 °C for 5 min. After precipitation in -20 °C overnight and centrifugation at 20,000 × g for 20 min at 4 °C, the supernatant was collected and stored at -80 °C for future analysis. Pellets were suspended in 0.2 M NaOH in 95 °C for 30 min, centrifuged at 20,000 × g for 20 min, and proteins in supernatant were then collected and quantified using the BCA kit (Cat. #23227, Thermo Scientific, USA) for normalization using protein content. α-KG (Cat. #K812223, Mcklin, China), α-ketoisocaproate (KIC, Cat. #M813122, Mcklin, China), α-keto-beta-methylvalerate (KMV, Cat. # M863189, Mcklin, China), α-ketoisovalerate (KIV, Cat. #S67930, YuanYe, China), succinate (Cat. #S817846, Mcklin, China), glutmate (Cat. #1294976, Sigma Aldrich, USA), glutamine (Cat. #D807113, Mcklin, China), leucine (Cat. #L037500, Sigma Aldrich, USA), isoleucine (Cat. #I0460000, Sigma Aldrich, USA), valine (Cat. #IV0030000, Sigma Aldrich, USA) were used to generate the quantitative standard curve of each metabolite. To assess recovery rates during sample derivation and the variation across replicates, samples were spiked with isotope-labeled α-ketoglutaric acid-3,3,4,4-D4 (Cat. #DLM-6201, Cambridge Isotope Laboratories, USA; final concentration of 200 ng/mL) and a mixture of ^13^C-labeled amino acids including valine, leucine, isoleucine, glutamate, glutamine (Cat. #MSK-A2-1.2, Cambridge Isotope Laboratories, USA; final concentration of 125 nmol/mL). For the analysis of all organic acids, 50 μL of the supernatant was dried under nitrogen. α-KG and ketoacids were derived as butyl esters as described 15. The samples were dried under nitrogen and reconstituted in 100 μL mobile phase.

Organic acids and amino acids were analyzed by HPLC-MS/MS using a Shimadzu LC-20ACXR (Shimadzu, Japan) coupled with a Sciex 4500 triple quadruple mass spectrometry (Sciex, USA) with heated electrospray ionization (ESI). For amino acids, ESI-MS was operated in positive mode with voltage set at 3500 V, temperature at 550 °C, and curtain gas pressure at 20 psi. Quantification was performed using multiple reaction monitoring (MRM) strategy: 204>84 for Glu, 203>83 for Gln, 188>86 for Leu and Ile. Reconstituted samples were introduced into LC -MS/MS with 5 μL injection using the built-in autosampler. A Phenomenex Kinetex C18 column (100 × 2.1 mm, 2.6 μm) was maintained at 40 °C. Solvent A was comprised of 100% H_2_O with 0.1% formic acid and solvent B was comprised of 100% acetonitrile (ACN) with 0.1% formic acid. The flow rate was 0.4 mL/min and the gradient was 20% solvent B at 0 min, maintaining 20% solvent B until 2.85 min, then increasing solvent B to 80% at 2.86 min, maintaining 80% solvent B until 4.2 min, then reducing to 20% solvent B at 6.0 min. For organic acids, the ESI-MS was operated in negative mode with voltage of -4500 V, a temperature of 500 °C, and a curtain gas pressure of 25 psi. The MRM transitions were α-KG 259>101, SA 117>73, KMV and KIC 129>129, KIV 115>115. A Phenomenex Kinetex PS-C18 column (50 × 2.1 mm, 2.6 μm) was maintained at 40 °C. The flow rate was 0.5 mL/min and the gradient was 0% B at 0 min, increasing to 50% B at 3.5 min, reducing to 0% B at 3.51 min, and then maintaining 0% B for another 2 min.

### Detection of global DNA methylation

Detection of DNA methylation levels was adapted from a previously described protocol 16. Cells were lysed in buffer (20 mM Tris-HCl pH 8.0, 10 mM NaCl, 10 mM EDTA pH 8.0, 0.5% SDS) at 37 °C overnight, then 100 μg/mL proteinase K was added and allowed to digest proteins at 60 °C overnight. DNA was extracted using phenol:chloroform:isoamyl alcohol 25:24:1 (Cat. #BSA03M1, BioFlux) and chloroform, precipitated in isopropanol at -20 °C for 30 min, then digested by nuclease P1 (Cat. #M0660S, NEB, UK) and dephosphorylated by antarctic phosphatase (Cat. #M289S, NEB, UK) at 37 °C for 4 h. 5-methyl-deoxycytosine and deoxycytosine were detected with 280 nm UV by an HPLC system (Agilent, 1260 Infinity II, USA) with a BEH C18 column (Cat. #186003625, Waters XBridge, 5 μm, 250 mm × 4.6 mm, USA). Separation was achieved at a flow rate of 0.5 mL/min using the following linear gradient: 0%-70% buffer B (100% MeOH) for 25 min, 70%-0% buffer B for 20 min. Buffer A was composed of 10 mM KH_2_PO_4_, pH 3.6. Nucleotides were analyzed by OpenLAB CDS ChemStation based on peaks areas.

### Western blot

Western blot analysis was performed as previously reported 13. The primary antibodies used were: anti-BCAT1 (1:1000, Cat. #TA504360S, OriGene, USA), anti-SOX2 (1:1000, Cat. #ab97959, Abcam, UK), anti-ACTB/β-actin (1:5000, Cat. #30101-ES10, YEASEN, China), anti-CPS1 (1:1000, Cat. #ab45956, Abcam, UK), anti-histone H3 (1:10000, Cat. #17168-1-AP, Proteintech, USA), anti-histone H3 (mono methyl K4) (1:3000, Cat. #ab8895, Abcam, UK), anti-histone H3 (di-methyl K9) (1:3000, Cat. #mab1220, Abcam, UK) and anti-histone H3 (tri-methyl K4) (1:3000, Cat. #ab8580, Abcam, UK). For one blot used to detect multiple proteins, anti-SOX2 and anti- ACTB/β-actin were added in 1% BSA TBS buffer containing 0.1% Tween-20 (1:1000) and incubated overnight at 4 °C. After 3 times washing, Alexa Fluor 790 AffiniPure goat anti-mouse IgG (H+L) (1:10000, Cat. #33219ES60, YEASEN, China) or anti-rabbit (1:10000, Cat. #33119ES60, YEASEN, China) secondary antibody was used.

### RNA extraction, real-time PCR

Total RNA was extracted using TRIzol. cDNA was synthesized using the Prime Script 1st Strand cDNA synthesis Kit (Cat. #6110A, Takara, Japan) for detection of SOX2, OCT4, NANOG. Transcript expression levels were normalized to B2M mRNA. cDNA of miR200 and U6 snRNA were synthesized using the miRNA 1st Strand cDNA synthesis Kit (Cat. #MR101-02, Vazyme, China). Quantitative PCR was performed on a real-time PCR system using SYBR Premix (Cat. #A6001, Promega, USA) and miRNA Universal SYBR qPCR Master Mix (Cat. #MQ101-02, Vazyme). Primer sequences are listed in Supplementary Table S1.

### Immunofluorescence microscopy

Immunofluorescence and image acquisition methods were performed as previously described 13.

### Trans-well migration assay

5 × 10^4^ cells in 100 μL serum-free medium were plated in the upper chamber of trans-well inserts (Cat. #3428, CORNING, USA) and cultured in medium containing 10% fetal bovin serum for 36 h. Migrated cells were fixed by 4% paraformaldehyde and stained with 0.5% crystal violet, then imaged for quantitative analysis.

### Flow cytometry and viability assay

Flow cytometry analysis was performed as previously described 13. Cells were labeled using a phycoerythrin (PE)-conjugated anti-CD133/2 antibody (1:50, 293C3 clone, Cat. #130-113-748, Miltenyi Biotech, Germany), and co-stained with 7-ADD (1:20, Cat. #559925, BD Pharmingen, USA) to label dead cells. Cells were analyzed using a BD LSRFortessa Flow Cytometer (BD Biosciences, USA) at the Flow Cytometry Core Facility of the School of Life Sciences, ECNU. The data were processed using the FlowJo program.

To detect apoptosis, cells were stained using a PE Annexin V Apoptosis Detection Kit (Cat. #559763, BD Pharmingen, USA). To assay cell viability, cells were reacted with the CCK8 kit (Cat. #40203ES76, YEASEN, China); OD values were measured at a wavelength of 450 nm and normalized to OD values of cells seeded after 24 h. Student's *t*-test was applied to compare the relative growth rate between cells measured at the same time point.

### Micro-CT imaging

Bones were scanned by micro-CT (Skyscan 1272, Bruker). The scanner was set at a voltage of 60 kV, a current of 166 µA and a resolution of 9 µm per pixel and the results were analyzed according to the manufacturer's instructions. Region-of-interest (ROI) was defined from 0.215 mm (12 image slices) to 1.72 mm (106 image slices), where the growth plate slice was defined as 0 mm. Contrast was defined from 65-255; 3D analysis, BMD and 3D models were analyzed using CTAn software (Bruker microCT). 3D models were adjusted in CTVol software (Bruker microCT).

### H&E staining

Bone tissues were fixed with 4% PFA for two days, decalcified with EDTA for two weeks until the tissues were soft, followed by dehydration, clearing, and embedding in paraffin. Tissues were cut in 4 mm sections followed by H&E staining. Sections were dewaxed and rehydrated, stained with hematoxylin and eosin according to manufacturer's instructions.

## Results

### SILAC-based quantitative proteomic comparison of primary and metastatic A549 cells

**Figure 1:**
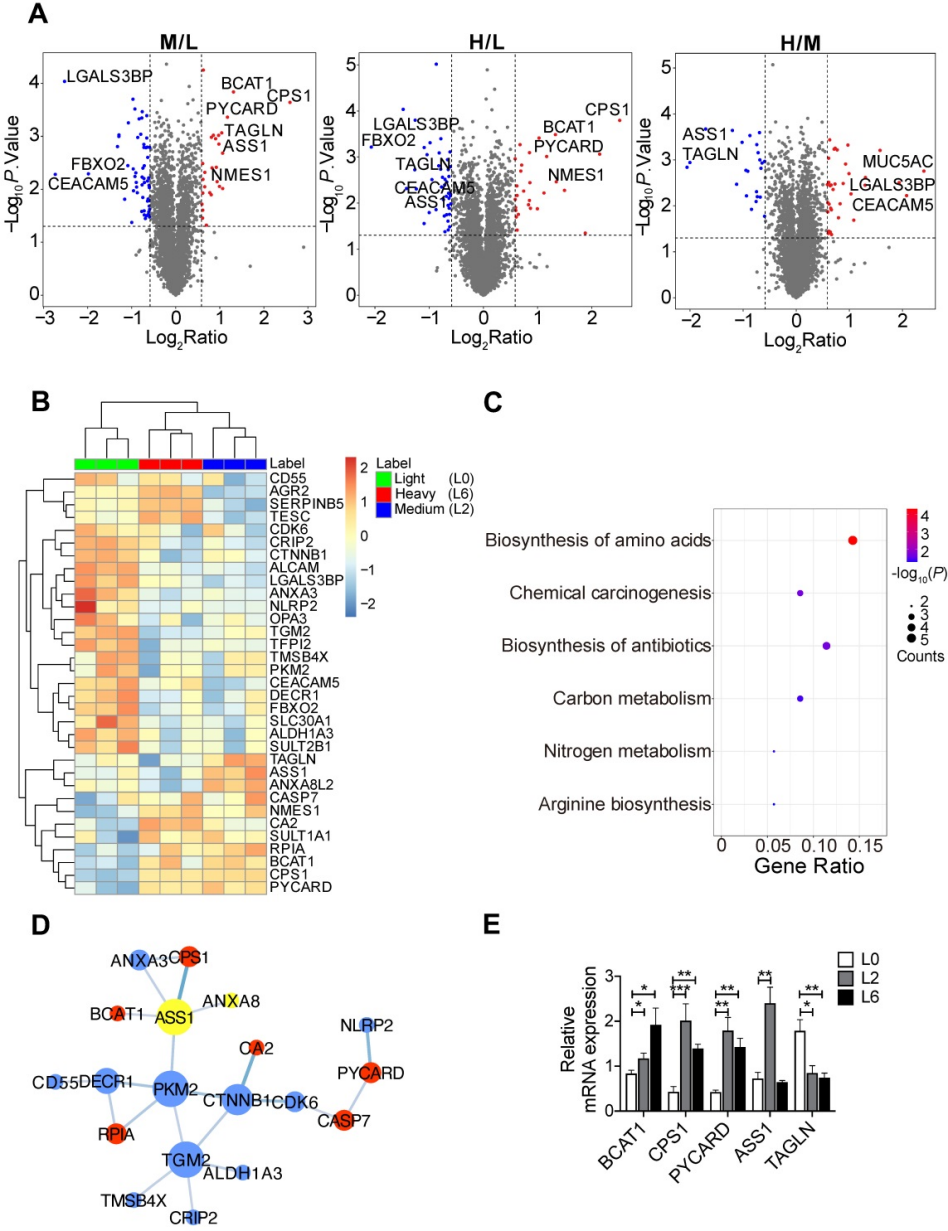
SILAC-based quantitative proteomic analysis of primary and metastatic A549 cells. **(A)** Volcano plots of SILAC ratios between two groups. Red and blue dots indicate significantly increased and decreased proteins, respectively. FC > 1.5 or FC < 0.67, *P* < 0.05. **(B)** Heatmap of significantly changed proteins in both L2 and L6 cells. Log_2_ proteins intensities were scaled and clustered using hierarchical clustering. **(C)** KEGG pathway analysis of proteins changed in both L2 and L6 cells. **(D)** Protein-protein interaction network of significantly changed proteins in L2 and L6 cells. Red and blue represent increase and decrease in both L2 and L6, yellow represents proteins with opposite direction of change. **(E)** Real-time PCR analysis of mRNAs of a subset significantly changed proteins. Student's *t*-test, *P < 0.05, ** P < 0.01, *** P < 0.001, n = 4.

To identify proteome changes associated with the metastatic state in lung cancer, we applied quantitative mass spectrometry analysis based on stable isotopic labeling of amino acids in cell culture (SILAC) (Figure S1A). We utilized the primary lung adenocarcinoma A549 cell line (designated L0), and L0 cells that underwent three rounds of *in vivo* selection, giving rise to spine metastatic cells (designated L2 and L6) 17. We cultured L0 cells in “light (Lys^0^Arg^0^)” medium, L2 cells in “medium heavy (Lys^4^Arg^6^)” medium, and L6 cells in “heavy (Lys^8^Arg^10^)” medium. After labeling, the three lines of cells were mixed and analyzed together by quantitative mass spectrometry. A total of 4453 proteins were quantified, among which 3796 proteins were quantified in all three biological repeats (Figure S1B, Table S2). Principal component analysis revealed good separation among the three cell lines (Figure S1C), with the first component explaining nearly 40% of the variation, indicating that the metastatic cells displayed a distinct protein expression profile compared to the primary cells. Relative to L0 cells, 74 proteins in L6 cells and 86 proteins in L2 were significantly changed, as shown in the volcano plots presented in Figure 1A. Extracted chromatograms of unique peptides from representative proteins are displayed in Figure S1D.

Among the significantly changed proteins, 33 were shared in both metastatic cell lines (L2 and L6) and are shown in a heatmap (Figure 1B); hierarchical clustering clearly separated the three sample groups. KEGG pathway analysis followed by protein-protein interaction network construction showed that the most significantly enriched pathway was amino acid metabolic process (Figure 1C-D, S2A-B). Among the node proteins, BCAT1, the rate limiting enzyme for BCAA metabolism, was the most consistently upregulated protein in metastatic cells. All the enzymes involved in BCAA metabolism were plotted in another heatmap (Figure S1E). Consistent with a report that most of the enzymes in BCAA metabolism were decreased in HCC tumors 18, most of the 23 enzymes quantified in our proteomic study were down regulated, except BCAT1, HADHB, and ACAA2. We then measured the mRNA levels of BCAT1 and other upregulated proteins - CPS1 and PYCARD - in both L2 and L6 cells, as well as ASS1 and TAGLN that showed an opposite direction of change in L2 and L6 cells (Figure 1E). The mRNA of BCAT1, CPS1 and PYCARD all increased in L2 and L6 cells, consistent with the changes measured at the protein level. While the mRNA of ASS1 increased only in L2 cells, TAGLN decreased in both metastatic lines. Upregulation of these proteins has been associated with multiple other cancer types 10, 19-22, indicating the validity of our data. CPS1 and ASS1 play an essential role in the urea cycle and deficiency of ASS1 promoted tumor proliferation in several cancers 23, 24. TAGLN is an actin-binding protein known to be upregulated by the TP53 and PTEN pathways 25. The role of TAGLN in different types of cancer is complicated. Its upregulation in bladder cancer was associated with poor prognosis 26, while its low expression promoted tumor invasion in colorectal cancer 27.

### Comparison of primary and metastatic tumor tissues from lung cancer patients

**Figure 2:**
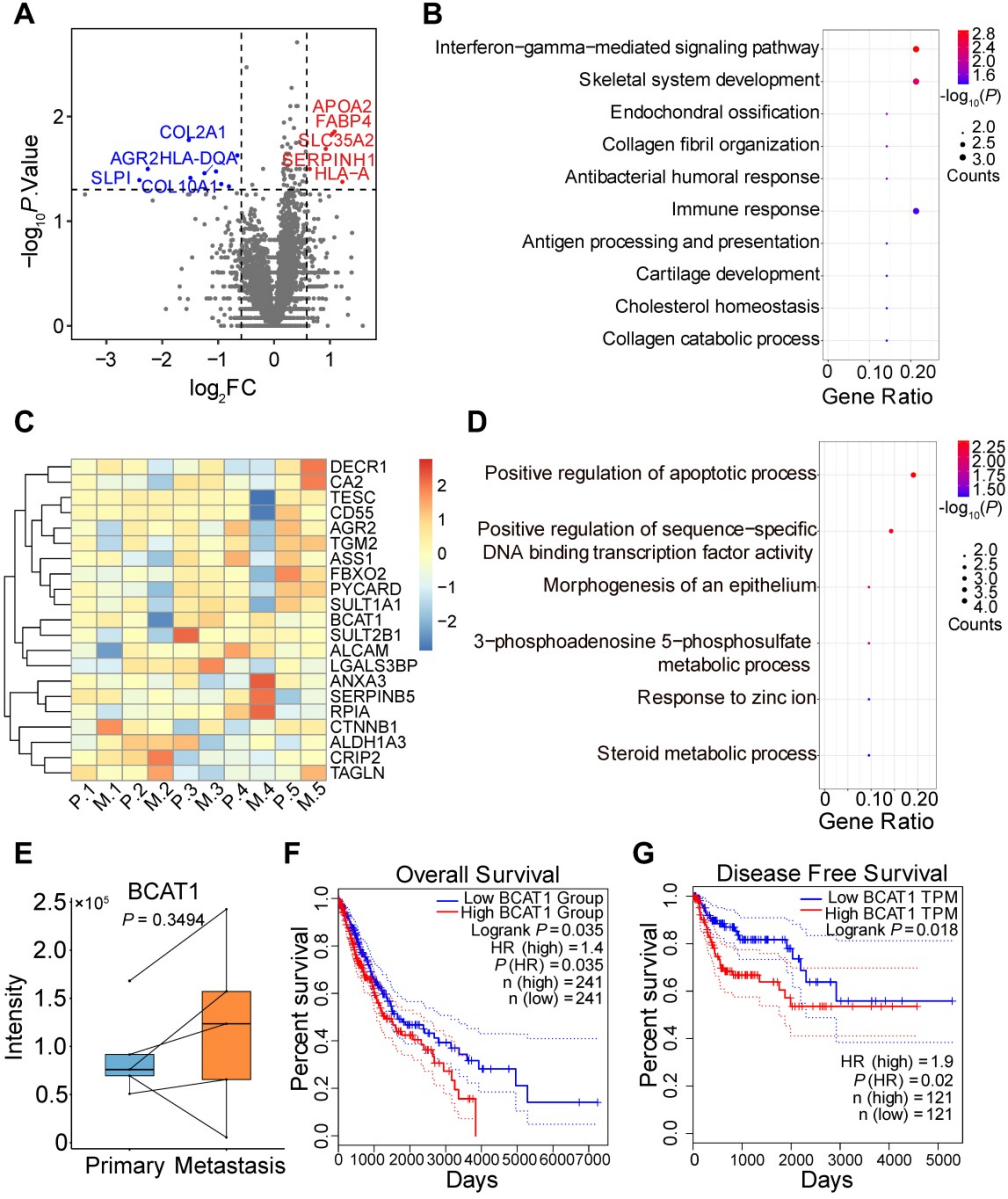
TMT-based quantitative proteomic analysis of primary and metastatic human tumor tissues. **(A)** Volcano plot of TMT-based quantitative proteomic results of tumor tissues from lung cancer patient tumor specimens. Tissues from primary and metastatic lung cancer of the same patient were paired. **(B)** Gene ontology analysis of the 14 significantly changed proteins from TMT proteomic analysis. **(C)** Heatmap of tissue protein expression profile of 21 proteins significantly changed in SILAC proteomics. **(D)** Gene ontology analysis of (C). **(E)** BCAT1 protein expression in primary and metastasis tissues samples from lung cancer patients (Paired student's *t*-test, n = 5). **(F)** Kaplan-Meier OS curve of LUAD and LUSC patients stratified by BCAT1 expression from the TCGA datasets. **(G)** Kaplan-Meier DFS curve generated from the LUSC dataset.

We went on to obtain four pairs of FFPE primary and metastatic tumor tissues for proteomic analysis, with each pair coming from the same patient. We included an additional pair of primary and metastatic frozen tissues from different patients for the analysis. Tandem mass tag (TMT) labeling and quantitative mass spectrometry analysis were applied to measure protein expression. We quantified 4799 proteins and identified 14 significantly changed proteins (Figure 2A), with quantitative results listed in Table S3 and respective clinical information listed in Table S4. Gene Ontology and KEGG enrichment analysis revealed that most of the changed proteins were involved in immune response pathways, including interferon-gamma and PPAR signaling (Figure 2B, S2C). The proteins involved in these pathways were TRIM68 28, FABP4 29, and APOA 30. In addition, we identified dysregulation of AGR2 in both SILAC and TMT datasets, which has been shown to promote cancer progression in multiple cancer types 31.

Among the significantly changed proteins in our SILAC data that were also quantified by TMT (Figure 2C), only BCAT1 displayed an increased trend of expression (but not statistically significant) in metastatic tissues (Figure 2E). Biology pathways of proteins listed in Figure 2C are shown in Figure 2D and S2D. We also examined the expression of BCAT1 from publicly available datasets 5, 32, 33 and found that BCAT1 was expressed at higher levels (but not statistically significant presumably due to small sample size) in metastatic tissues compared to primary tissues in both proteomic and transcriptomic data (Figure S3A). To gain additional insights on the potential role of BCAT1 dysregulation in lung cancer, we utilized TCGA data and performed Kaplan-Meier survival analysis based on mRNA expression of BCAT1. In LUAD and LUSC datasets, the overall survival (OS, n = 241) of patients were negatively correlated with the expression of BCAT1, with a hazard ratio of 1.4 and *P* value less than 0.05 (Figure 2F). The disease-free survival (DFS, n = 241) of the same dataset displayed a hazard ratio of 1.3 without statistical significance (Figure S3B). It also appeared that in the LUSC dataset, DFS (n = 121) negatively correlated with BCAT1 expression with a hazard ratio of 1.9 and with statistical significance (Figure 2G). The OS (n = 121) also exhibited the same trend without statistical significance (Figure S3C). The same survival analysis using the proteomic data from Xu et al. 4 (Figure S3D-E) did not show statistical significance either. These results indicated that BCAT1 might be a weak prognostic marker for lung cancer progression.

### High expression of BCAT1 promotes metastasis of cancer cells

**Figure 3:**
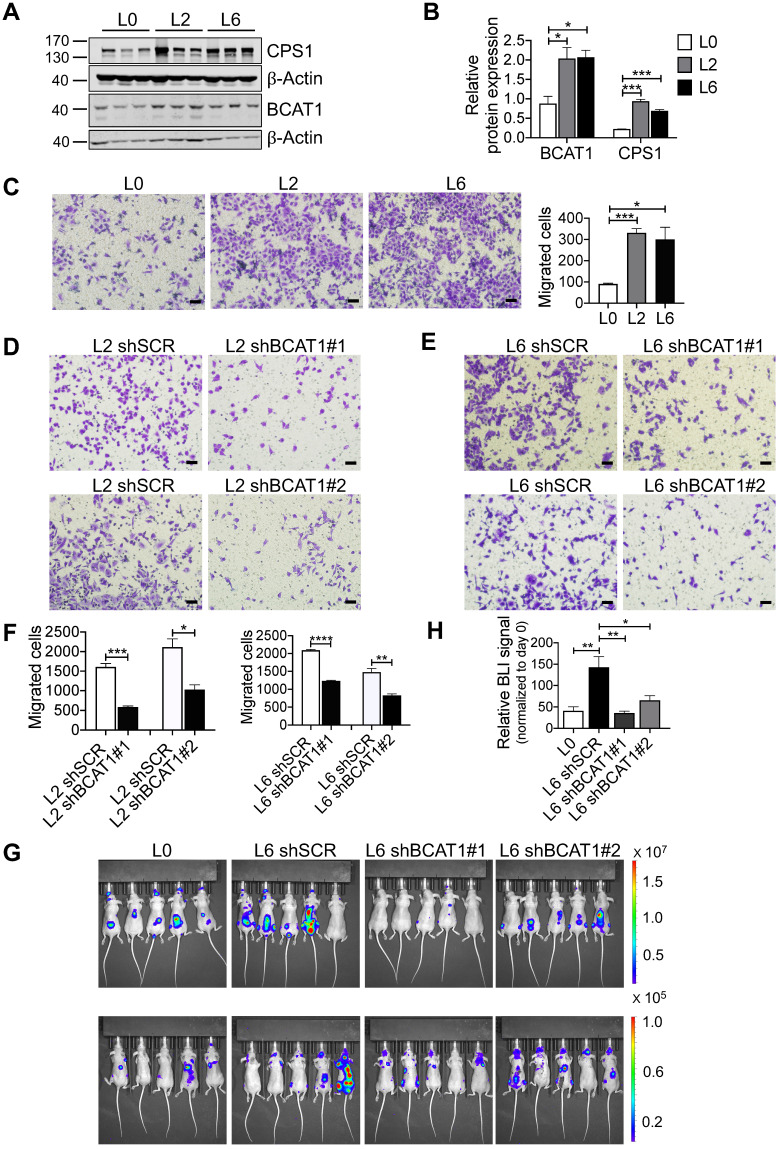

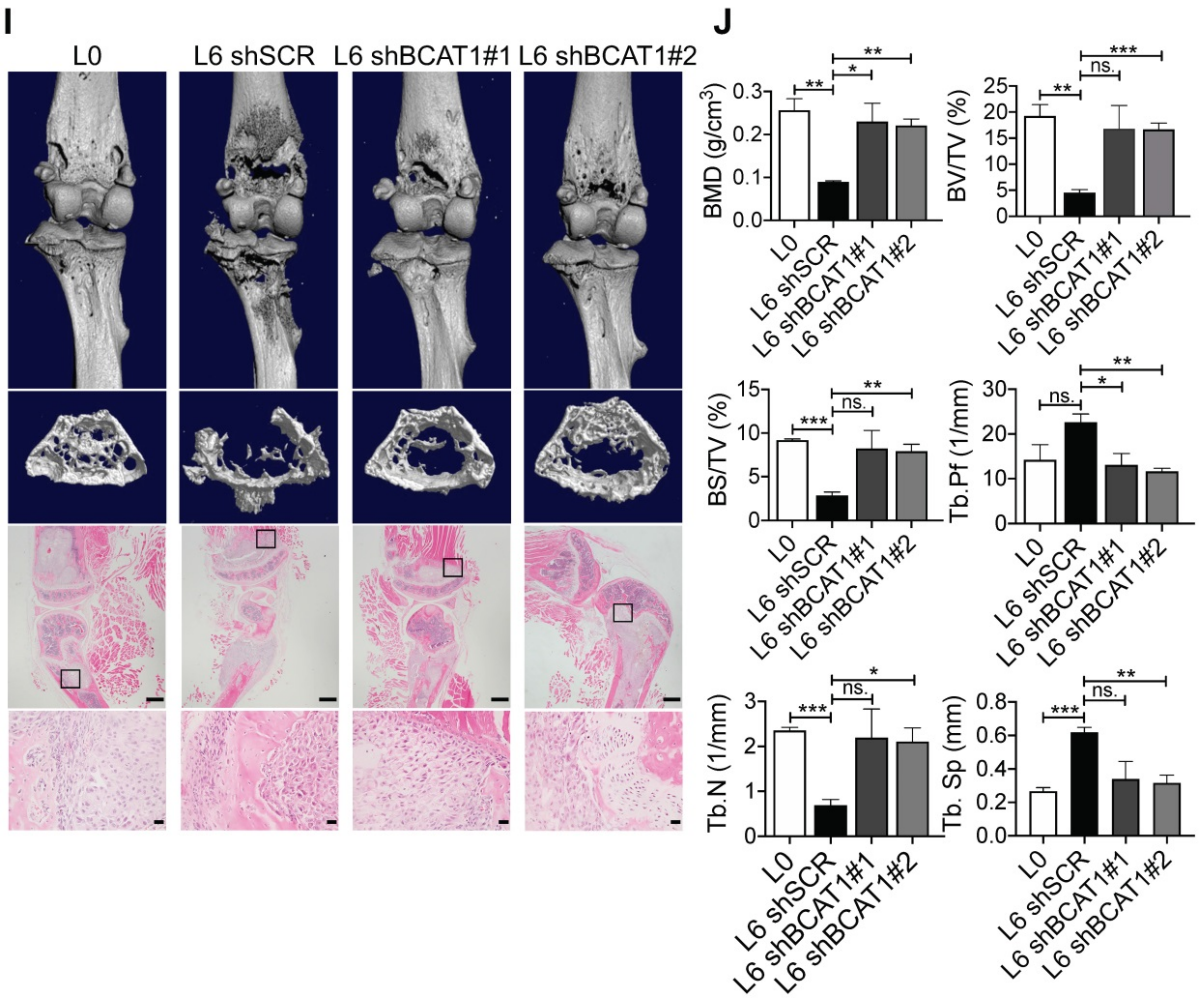
High expression of BCAT1 promotes cancer cell migration and metastasis.** (A)** Western blot analysis of BCAT1 and CPS1 expression in primary and metastatic A549 cells. **(B)** Statistical analysis of the Western blot results in (A) (Student's *t*-test, *P < 0.05, ** P < 0.01, *** P < 0.001, n = 3). **(C)** Representative images (36 h after seeding) and bar graph of associated group data from trans-well studies of L2 and L6 cell migration (magnification: 200 ×, scale bar: 50 μm, n = 3). **(D, E)** Images from trans-well assays showing decreased migration of L2 and L6 cells with stable knockdown of BCAT1 (magnification: 200 ×, scale bar: 50 μm). **(F)** Bar graphs of group data from trans-well studies of the effect of BCAT1 knockdown on L2 and L6 cell migration. *P < 0.05, ***P < 0.001, n = 3. **(G)** Representative IVIS images of tumor growth and metastasis.** (H)** Bar graph showing the relative bioluminescence intensity (BLI) of 4 groups; the data were analyzed using Kolmogorov-Smirnov test since the data was not normally distributed. * *P* < 0.05, n = 10. To exclude the difference of luciferase intensities in day 0 injection, we normalized BLI signals to respective signals in day 0. **(I)** Representative mCT images of femurs from the mice showing the proximal femur and trabecular bone of the femur metaphysis, as well as H&E staining for tumor site of origin (magnification: 20 ×, scale bar: 500 μm, and 400 ×, scale bar: 20 μm). **(J)** Bar graph of quantitative mCT analysis of trabecular bone parameters of femurs from the mice. BMD, bone mineral density; BV/TV, percent bone volume; BS/TV, bone surface density; Tb. N, trabecular number; Tb. Sp, trabecular separation; Tb. Pf, trabecular pattern surface.

Consistent with our SILAC proteomic results using primary and metastatic A549 cell lines, we assessed the expression of BCAT1 and CPS1 by Western blot and found that both were significantly increased in metastatic cells (Figure 3A-B). CPS1 is a mitochondrial enzyme catalyzing the synthesis of carbamoyl phosphate from ammonia and bicarbonate, and has been shown to increase in lung adenocarcinoma tissues 19. However, as only BCAT1 expression showed evidence of upregulation in patient samples, we focus our attention on BCAT1.

To explore the potential role of BCAT1 in lung cancer cell metastasis, we first examined cell migration. Using a standard trans-well assay, we observed that the metastatic L2 and L6 cells showed significantly higher migratory capacity than the primary L0 cells (Figure 3C). We then generated L2 and L6 cells stably expressing shRNA against BCAT1 (shBCAT1) and performed the same trans-well assay. Migration of cells expressing shBCAT1 were significantly slower than control cells expressing scrambled shRNA (Figure 3D-F). To rule out a potential confounding influence of cell growth or death due to BCAT1 knock down, we assayed for proliferation and apoptosis. As shown in Figure S4A-C, reducing BCAT1 expression had minimal influence on apoptosis, whereas the proliferation were slightly decreased after 48 hours.

To determine whether BCAT1 modulates metastasis *in vivo*, we intracardially inoculated cancer cells stably expressing either shBCAT1 or control shRNA into nude mice, and examined metastasis using bioluminescence imaging. Mice inoculated with L6 cells expressing shBCAT1 showed decreased cancer cell metastasis compared to control cells (Figure 3G-H). Micro-CT analysis of the major metastatic sites, the femur and the tibia, revealed severe osseous erosion in mice inoculated with metastatic cells (compare L6 to L0 cells), whereas mice inoculated with metastatic cells expressing shBCAT1 showed much less osseous erosion (Figure 3I). A quantitative analysis of CT images of the bone is displayed in Figure 3J. Overall, knocking down BCAT1 impaired the migration of metastatic L2 and L6 cells *in vitro* and greatly reduced the severity of bone metastasis *in vivo*, almost to that of the L0 level. Taken together, it appears that overexpression of BCAT1 may be a causal event driving lung cancer cell metastasis, and reducing BCAT1 expression suppressed cancer cell migration and metastasis.

### BCAT1 promotes metastasis through increasing the expression of stemness factors

**Figure 4:**
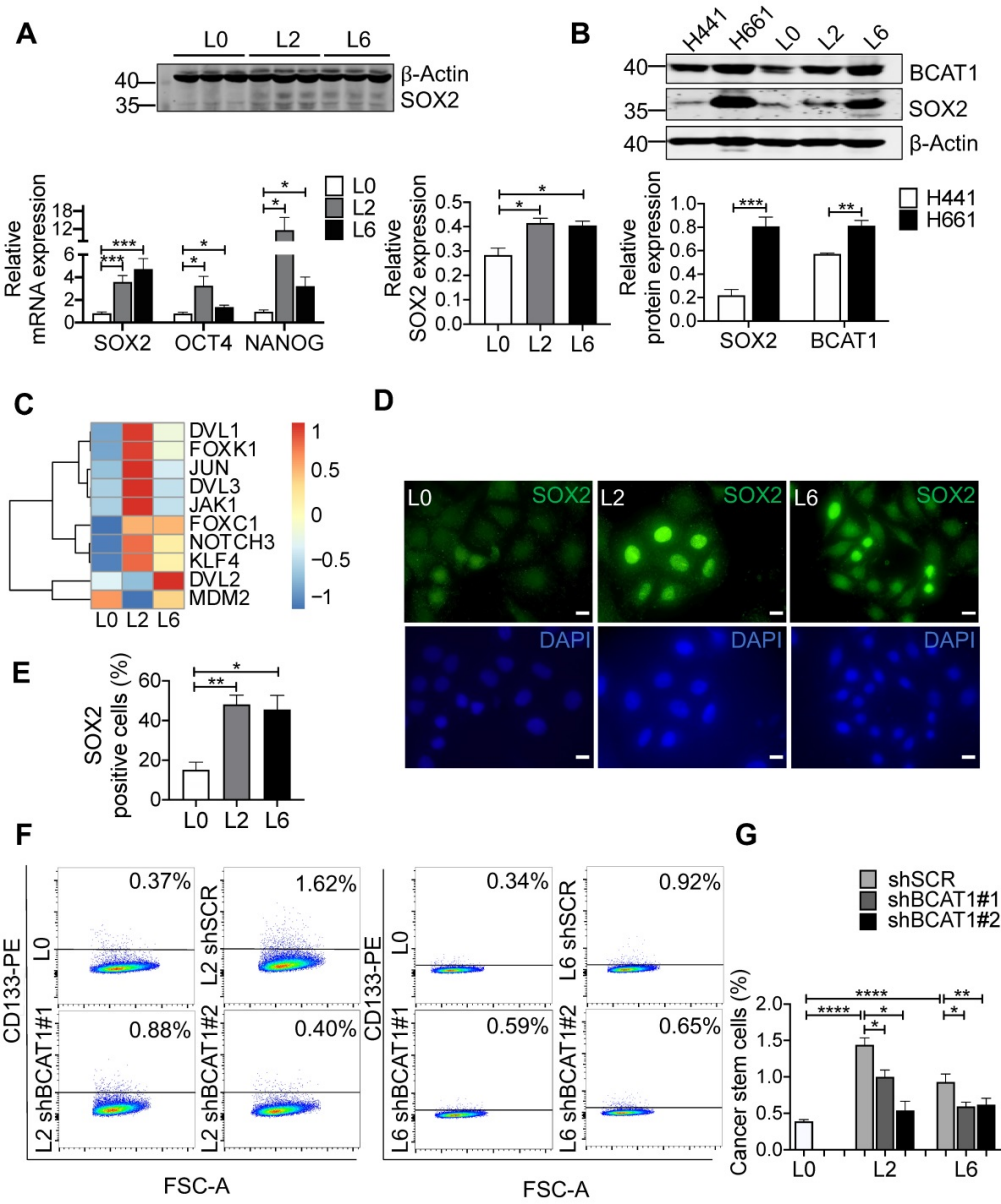
Upregulation of stemness-associated factors in metastatic cells. **(A)** Western blot analysis of SOX2 (n = 3) and real-time PCR analysis (n = 6) of three stemness factors (Student's *t*-test, * *P* < 0.05, ** P < 0.01, *** P < 0.001). **(B)** Western blot analysis of BCAT1 and SOX2 in human primary lung cancer cells (H441) and lung cancer cells isolated from lymph node metastasis (H661), n = 3.** (C)** mRNA levels of SOX2-regulated genes from a previous RNA-seq data, n = 1.** (D)** Immunofluorescent imaging of SOX2 (green) and DAPI staining in L0, L2 and L6 A549 cells (magnification: 1000 ×, scale bar: 10 μm). **(E)** Bar graph of SOX2-positive cells from immunocytochemistry studies, n = 3. **(F)** FACS analysis of CD133 expression in control (L0) and L2 and L6 cells expressing shBCAT1. Cells were stained with CD133-PE, co-stained with 7-ADD. **(G)** Bar graph of FACS group data (Student's *t*-test of data from F). * *P* < 0.05, ** P < 0.01, **** P < 0.0001, n = 3~5.

To gain mechanistic insights into BCAT1-mediated metastasis, we examined the expression of genes associated with stemness and epithelial-to-mesenchymal transition (EMT), well-established cellular states that are associated with increased migration and metastasis. Stemness transcription factor SOX2 was significantly elevated in both L2 and L6 cells, at both the mRNA and protein level (Figure 4A). Increased expression of both BCAT1 and SOX2 were also observed in H661 cells which originated from lymph node metastasis of a patient with large cell carcinoma, compared to another primary lung cancer cell line H441 (Figure 4B).

SOX2 is a transcription factor maintaining the plasticity of embryonic stem cells and cancer stem cells, and overexpression of SOX2 promotes metastasis in lung cancer 34, 35. In our case, increased SOX2 expression may transform L0 cells into a poorly differentiated state with increased migratory potential. We re-analyzed the RNA-seq data of these A549 cells from a previous study 17, focusing on transcription factors. Many genes downstream of SOX2 were significantly upregulated in L2 and L6 cells, including Wnt signaling genes NOTCH3, DVL1, DVL2, and the oncogene KLF4 (Figure 4C). Consistent with a role for these Wnt factors in BCAT1-associated enhancement of lung cancer cell migration, a previous study reported that BCAT1 promoted lung cancer cell invasion and proliferation via activating Wnt signaling 10. However, Wnt signaling proteins including CTNNB1 and DVL2 were reduced in metastatic cells in our SILAC results (Figure S5A). A prior study by He et al. also showed that overexpression of SOX2 led to inhibition of Wnt/β-catenin signaling, potentially by upregulating GSK3β in A549 cells 36. On the other hand, transcription factors known to interact with SOX2, including FOXK1 and FOXC1 37, also increased expression (Figure 4C). We hypothesized that SOX2 inhibits Wnt signaling through β-catenin to sustain the undifferentiated state of metastatic cells.

**Figure 5:**
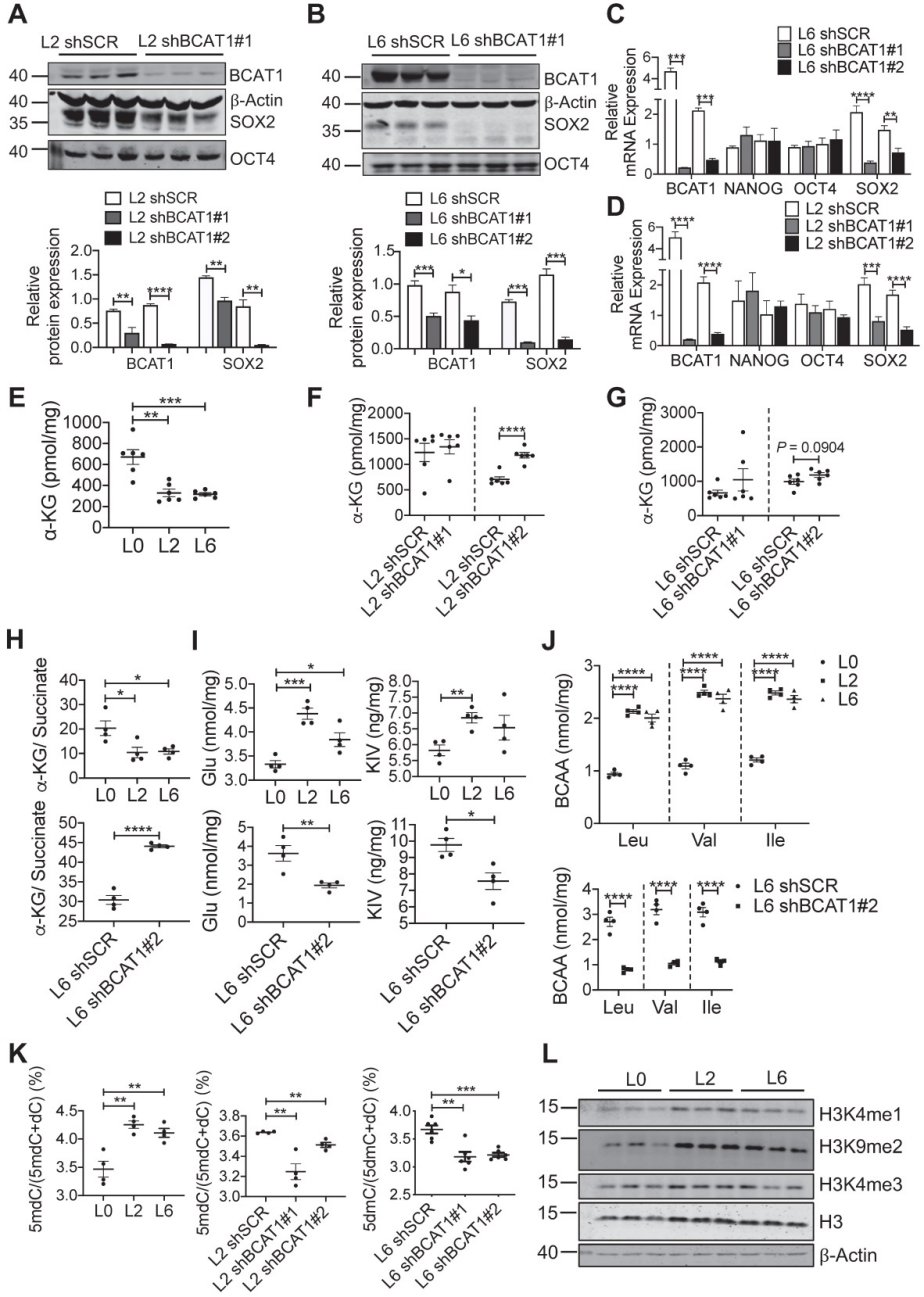
Reducing SOX2 expression by modulating BCAT1 levels. **(A, B)** Western blots and associated bar graphs showing reduced expression of SOX2 after knocking down BCAT1 (Student's *t*-test, * P < 0.05, n = 3). **(C, D)** Bar graphs showing the effect of BCAT1 knockdown on SOX2 transcription in L2 and L6 cells (n = 4~8). **(E, F and G)** Quantification of α-KG in A549 cells with or without BCAT1 knockdown, n = 6. **(H)** α-KG-to-succinate ratio, calculated using the measured concentrations from (E and G) and Figure S7A-B. **(I)** Quantification of glutmate and KIC in A549 cells, n = 4. **(J)** Quantification of BCAA in A549 cells, n = 4.** (K)** Quantification of 5-methyl-deoxycytosine (5mdC) and deoxycytosine (dC) in A549 cells, n = 4~6.** (L)** Western blot analysis of histone methylation in L0, L2 and L6 cells.

We utilized several approaches to validate SOX2 upregulation in metastatic A549 cells and to determine if this is linked upstream to BCAT1. Fluorescent imaging showed a higher proportion of SOX2-positive cells in L2 and L6 cells (Figure 4D-E). Flow cytometry analysis showed that CD133, a cancer cell stemness marker 38, was expressed in a higher proportion of L2 and L6 cells expressing scrambled BCAT1 shRNA, and that knockdown of BCAT1 expression significantly reduced the proportion of CD133-positive cells (Figure 4F-G). Furthermore, Western blot analysis showed that in L2 cells stably expressing shBCAT1, SOX2 was significantly reduced, while another stemness factor OCT4 showed no significant change (Figure 5A and Figure S5B). A similar effect on SOX2 was also observed in L6 cells (Figure 5B and Figure S5C). Furthermore, we demonstrated that knocking down BCAT1 reduces SOX2 expression in another metastatic lung cancer cell line H661 (Figure S5D). Real-time PCR showed consistent reduction of SOX2 mRNA in L2 and L6 cells stably expressing shBCAT1 (Figure 5C-D). These results indicate that BCAT1 promotes SOX2 expression in metastatic A549 cells and that this new pathway may be an important regulator of cell stemness in lung cancer.

Analysis of important EMT transcription factors showed that only ZEB1 displayed mild upregulation at mRNA level in L6 cells (Figure S6A), and E-cadherin protein was increased after knocking down BCAT1 (Figure S6B-F). In addition, we examined the correlation between BCAT1 and stemness- or EMT-related genes based on publicly available expression data 5, 32 (Figure S6G). In general, the correlation coefficients between two RNA-seq datasets showed better consistency than comparisons between RNA-seq and proteome datasets. Since EMT markers did not show consistent change, we speculate that BCAT1 may not have a strong influence on the EMT process.

### BCAT1 influences SOX2 expression through a microRNA mechanism

Increased BCAT1 expression may result in increased activity, thus depleting its substrate α-KG, which may have a regulatory impact on SOX2. Mass spectrometry measurement showed that α-KG levels were indeed reduced in L2 and L6 cells, and reducing BCAT1 expression partially restored α-KG levels (Figure 5E-G). Recent evidence indicates that an increased α-KG-to-succinate ratio is linked to chromatin modification and tumor cell differentiation, and contributes to p53-driven tumor suppression 39. We found that the α-KG-to-succinate ratio was decreased in metastatic cells, presumably due to the reduction of α-KG, and the ratio was recovered in BCAT1-knockdown cells with reduced succinate (Figure 5H and Figure S7A-B). On the other hand, glutmate, KIV, as well as BCAAs accumulated in metastatic cells (Figure 5I-J). KIC and its isomer KMV were decreased in L6 cells, which may be explained by the different usage of BCKAs. Reducing BCAT1 expression decreased the levels of these amino acids and keto acids (Figure 5I-J, Figure S7C-D). Meanwhile, glutamate concentration was reduced after inhibiting BCAT1 enzymatic activity using gabapentin (Figure S7E). Since α-KG is a cofactor of DNA demethylase TET2, reducing α-KG concentration may lead to hypermethylation of target genes at their promoter regions and thus silence these genes 40. We measured DNA methylation and found that 5-methyl deoxycytosine (5mdC) was significantly increased in L2 and L6 cells, indicating a trend of global DNA hypermethylation. In L2 and L6 cells expressing sh-BCAT1, 5mdC was largely reduced (Figure 5K). In addition, we observed increased histone methylation in L2 and L6 cells (Figure 5L).

**Figure 6:**
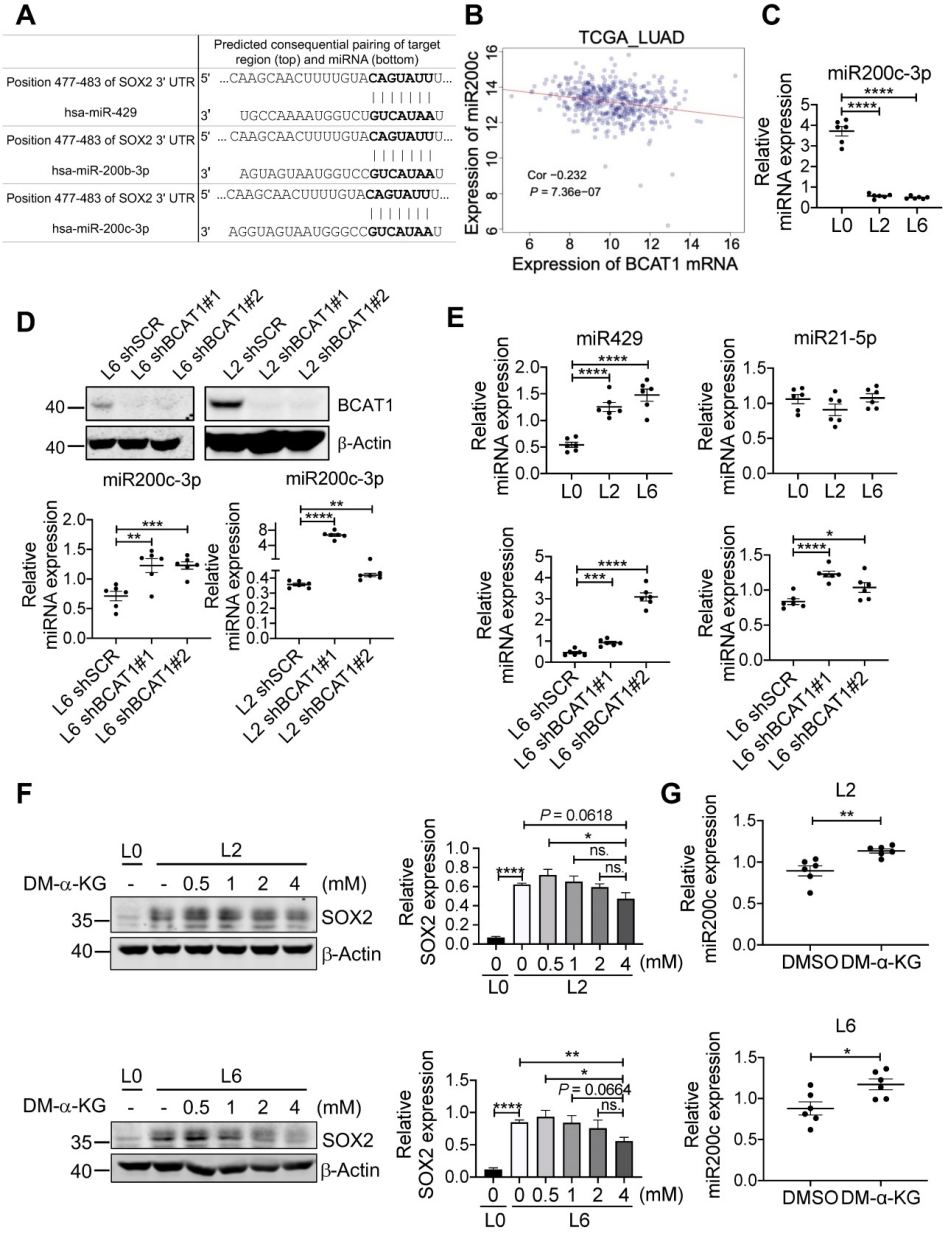
BCAT1 regulates the expression of miR200c and its target SOX2.** (A)** Target sequence of several microRNAs including miR200c at the 3'UTR of SOX2 gene (Targetscan.org).** (B)** Correlation analysis of BCAT1 mRNA and miR200c expression from TCGA LUAD data, correlation efficient (Cor) and *P* value are shown.** (C)** Expression of miR200c in A549 cells (* *P* < 0.05, n = 6).** (D)** Real-time PCR analysis of miR200c in L2 and L6 cells expressing shBCAT1 and scrambled shRNA, n = 6. The efficiency of BCAT1 knockdown is shown. **(E)** Expression of miR429 and miR21-5p in A549 cells with or without BCAT1 knocking down.** (F)** Effect of DM-α-KG treatment on SOX2 expression in L2 and L6 cells. The cells were treated with 4 mM DM-α-KG for 4 h.** (G)** Effect of DM-α-KG treatment (4 mM 4 h) on miR200c expression in L2 and L6 cells.

Among many genes regulated by TET2 are genes encoding micro-RNAs 41, and it has been shown that miR200 family members are negative regulators of SOX2 (Figure 6A) 42, 43. This raises the possibility that BCAT-mediated reduction of α-KG results in hypermethylation of miR200 family members and a lifting of translational inhibition of SOX2. Analysis of TCGA LUAD datasets revealed that the expression of miR200c was negatively correlated with BCAT1 (Figure 6B), and the negative correlation was also true for miR200a and miR429 (Figure S8A-B). Reduced expression of miR200a and miR429 correlated with poor OS and DFS of LUAD patients with statistical significance (Figure S8C-F), but miR200c showed no effect on patient survival (Figure S8G-H). Nevertheless, it has been reported that loss of miR-200c expression induces an aggressive phenotype in NSCLC 44. We measured miR200c and observed a large, statistically significantly reduction in L2 and L6 cells; moreover, knocking down BCAT1 increased miR200c expression (Figure 6C-D). The other two microRNAs that could target SOX2 mRNA, miR429 and miR21-5p, also displayed similar effect after BCAT1 knocking down, although they showed different levels of expression in metastatic cells (Figure 6E). If a BCAT1-α-KG-miR200c-SOX2 regulatory pathway holds true, we might be able to reduce SOX2 expression in metastatic cells via increasing α-KG concentration. Indeed, adding DM-α-KG, an α-KG analogue that is able to penetrate the cell membrane, reduced SOX2 expression in a dose-dependent manner in both L2 and L6 cells (Figure 6F). Treatment with DM-α-KG also increased miR200c in metastatic cells (Figure 6G). Taken together, our data indicates that BCAT1 plays an important role in driving lung cancer cell metastasis through modulating the expression of stemness factor SOX2 at the post-transcriptional level, and identify α-KG as a key signaling intermediate in this process.

## Discussion

Mounting evidence has linked overexpression of BCAT1 to tumor proliferation and progression 45. Many types of tumors reprogram BCAA metabolism, leading to accumulation of BCAAs 8, 11, 18, 45, 46, restriction of α-KG 47, activation of mTORC1 8, 18, 46, and promotion of hypoxia induced transcription and DNA hypermethylation 9, 47. However, whether BCAT1 plays a role in tumor metastasis is less well known. In this study, we demonstrate that overexpression of BCAT1 plays an important role in lung cancer cell migration and metastasis. From this perspective, our results potentially add a new contributing factor to the high mortality rate of lung cancer.

Our proteomic analysis also found that BCAT2, the mitochondrial isoform of BCAT1, was slightly decreased in L2 but not L6 cells (Table S2). The mild reduction of BCAT2 was also found in metastatic lung cancer tissues but without statistical significance due to variation between patients (Table S3). While loss of both BCAT1 and BCAT2 has been associated with impaired NSCLC tumor formation but no effect on pancreatic ductal adenocarcinoma (PDAC) 11, increasing expression of BCAT2 facilitated BCAA uptake and sustained mitochondrial respiration in PDAC 48. These seemingly conflicting results suggest complex regulatory mechanisms underlying BCAA metabolism in different tumor types. Since BCKDH, the BCKA dehydrogenase, is localized in the mitochondria, BCAAs transported into mitochondria are likely catabolized to participate in TCA cycle for energy supply. Cytoplasmic BCAAs, on the other hand, are primarily used as precursors for biosynthesis of non-essential amino acids and nucleotides. A recent study reported that several enzymes catalyzing irreversible reactions downstream of BCATs in BCAA metabolism, such as BCKDH, were reduced in different cancer types, and this reduced expression had significant impact on patient outcome 11, 18. Lower expression of BCAT2 might be an antagonistic signal for tumor cells that use BCAAs as nitrogen source.

SOX2 plays a major role in the maintenance of stemness and tumorigenicity in cancer stem cells and contributes to self-renewal and proliferation of stem-like side-population cells 49, 50. Increased expression of SOX2 has been observed in various cancers and associated with poor prognosis in NSCLC 50. As one of the four transcription factors that are sufficient to induce pluripotency 51, SOX2 regulates EMT through Wnt signaling in several cancer types including breast cancer 52 and colorectal cancer 53. To explore a possible causal relationship between overexpression of BCAT1 and increased SOX2 expression in metastatic lung cancer cells, we knocked down BCAT1 in these cells and observed consequent reduction of SOX2 at both the mRNA and protein level. Since cytoplasmic α-KG is mainly generated from IDH1 9, its level regulates DNA methylation through influencing the activity of dioxygenases such as TET family members, for which α-KG is a key cofactor 40. Depletion of BCAT1 results in α-KG accumulation in leukemia stem cells 47, which is consistent without observation that elevated expression of BCAT1 in metastatic A549 cells causes α-KG restriction. Thus, high BCAT1 level maintains SOX2 expression through reducing α-KG.

To explore how low α-KG level maintains high SOX2 expression, we examined the levels of miR200 family members (Figure 6), known negative regulators of many key transcription factors including SOX2 54. We found that high expression of BCAT1 negatively regulates miR200c expression in metastatic lung cancer cells, suggesting a possible post-transcriptional influence of BCAT1 on SOX2 expression. Lower α-KG levels result in reduced miR200c expression via lower TET activity and thus increased methylation on the promoter region of miR200c genes 41, 54, 55. This strongly suggests that the influence of BCAT1 on SOX2 expression involves α-KG as a key metabolic intermediate that regulates miR-200c transcription. Accordingly, we found that addition of exogenous α-KG decreased SOX2 and increased miR-200c in L2 and L6 metastatic A549 cells. We could not rule out the possibility that both SOX2 and miR200c had a parallel influence on EMT 52, 53. However, while our data showed consistent changes of epithelial marker E-cadherin following modulation of BCAT1 level, changes of the mesenchymal marker N-cadherin were insignificant under the same conditions. Follow up studies will be necessary to clarify whether BCAT1 is regulating EMT.

In conclusion, we found that BCAT1 expression was upregulated in metastatic lung cancer cells, regulates their migration and metastasis, and is associated with poor prognosis. In addition, our studies define a new pathway that involves α-KG as a key metabolic signaling intermediate between BCAT1 and the post-transcriptional regulation of SOX2 expression in metastatic lung cancer cells. These findings may open up new strategies to therapeutically target the metastatic process in lung cancer.

## Supplementary Material

Supplementary figures and tables 1 and 4.

Supplementary table 2.

Supplementary table 3.
