# Supplementary material for: Proteomic analysis of lung cancer cells reveals a critical role of BCAT1 in cancer cell metastasis
Source: Theranostics. 2021 Sep 27;11(19):9705–20. doi: 10.7150/thno.61731 (PMC8490523; doi:10.7150/thno.61731)
Supplement: Supplementary file 1 — Supplementary figures and tables 1 and 4. [file thnov11p9705s1.pdf]

Proteomic analysis of lung cancer cells reveals a critical role of BCAT1 in cancer cell metastasis

Lin Mao[1], Jin Chen[1], Xue Lu[1], Canlin Yang[2], Yi Ding[1], Mingming Wang[3], Yunpeng Zhang[1], Yuying Tian[1], Xing Li[2], Yunyun Fu[1], Yueying Yang[1], Yunyun Gu[3], Fei Gao[3], Junxing Huang[2], Lujian Liao[1*]

[1]Shanghai Key Laboratory of Regulatory Biology, School of Life Sciences, East China Normal University, Shanghai 200241, China.
[2]Department of Oncology, Taizhou People's Hospital, Affiliated to Nanjing University of Chinese Medicine, Taizhou, Jiangsu 225300, China
[3]Durbrain Medical Laboratory, Hangzhou, Zhejiang 310000, China.

[*]Corresponding author: ljliao@bio.ecnu.edu.cn

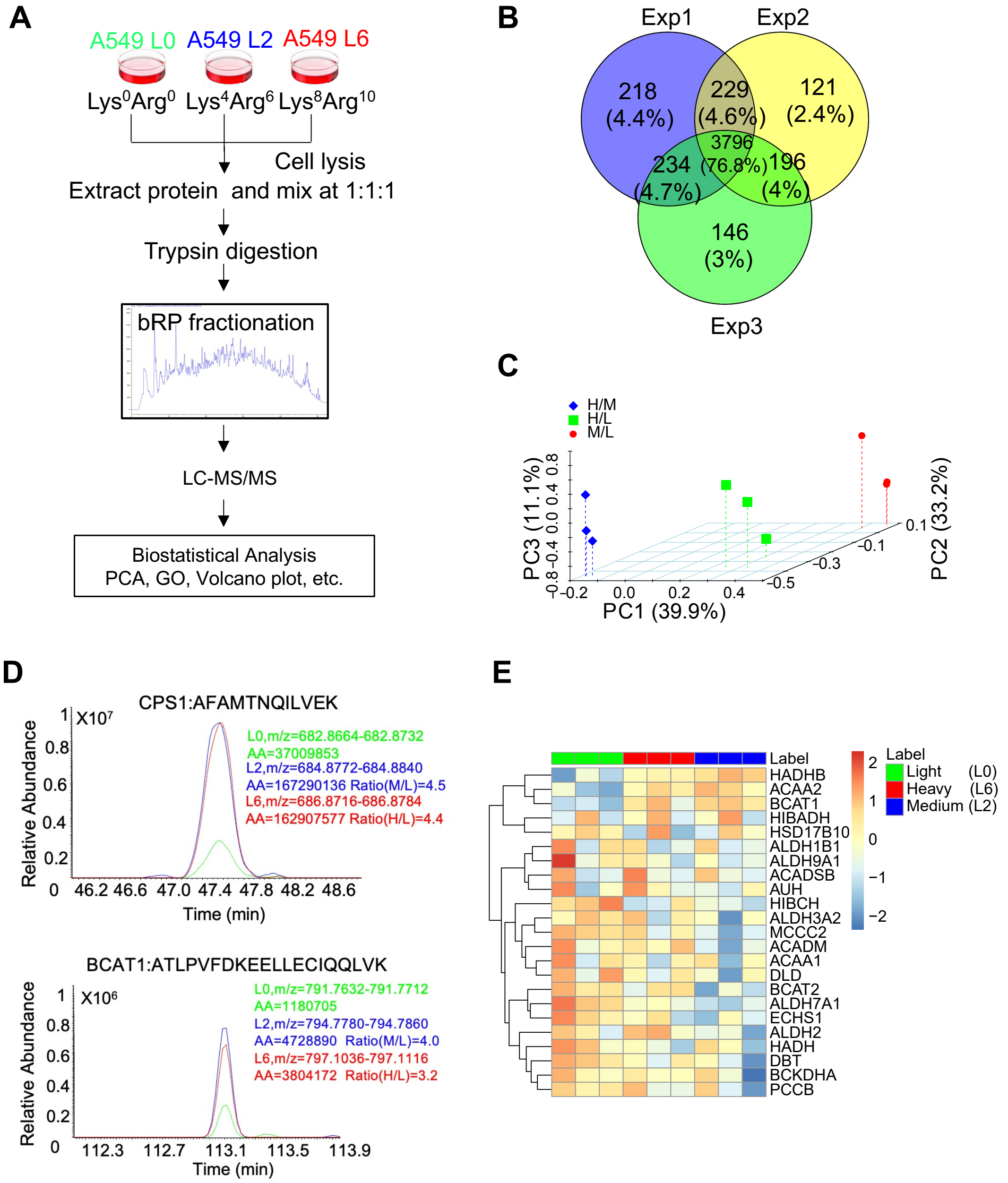

Figure S1. Reproducibility and accuracy of SILAC-based quantitative proteomics. **(A)** Schematic workflow of triple-labeling SILAC. **(B)** Overlaps of quantified proteins in three biological replicates. **(C)** Principal component analysis of SILAC ratio, L: light (L0); M: medium (L2); H: heavy (L6). **(D)** Reconstructed chromatograms of unique peptides of CPS1 and BCAT1 in L2 (blue) and L6 (red) cells. **(E)** The expression of enzymes involved in BCAA metabolism.

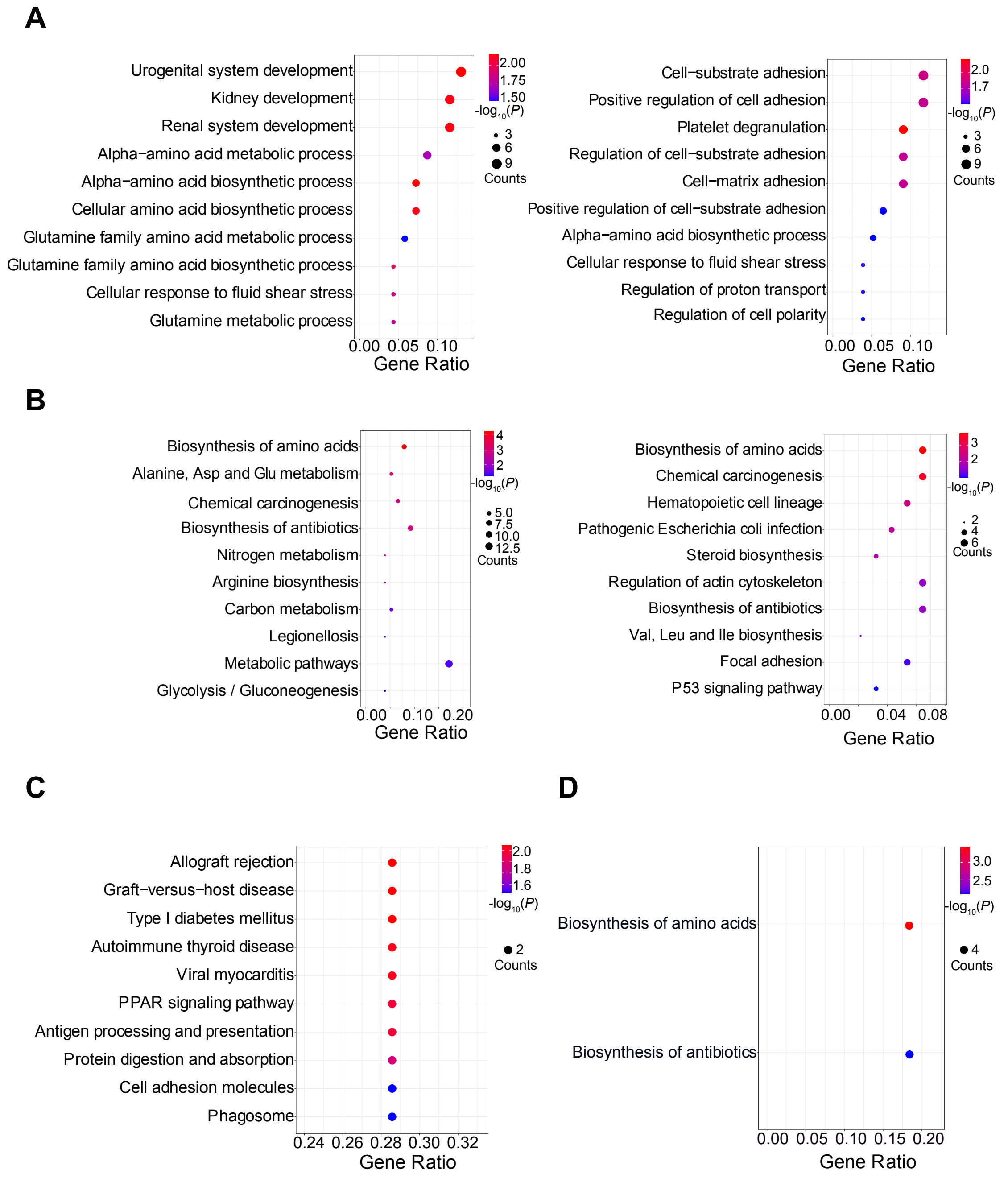

Figure S2. Enrichment analysis of differentially expressed proteins. **(A)** Gene ontology enrichment analysis of significantly changed proteins in heavy (L6) and medium (L2) comparing to light (L0) cells. Fold change > 1.5 or < 0.67 was used as the cutoff. $P < 0.05$. **(B)** KEGG pathway analysis of significantly changed proteins. **(C)** KEGG pathway analysis of significantly changed proteins from patient tissue samples. **(D)** KEGG pathway analysis of 21 proteins significantly changed in cell line and quantified in human samples.

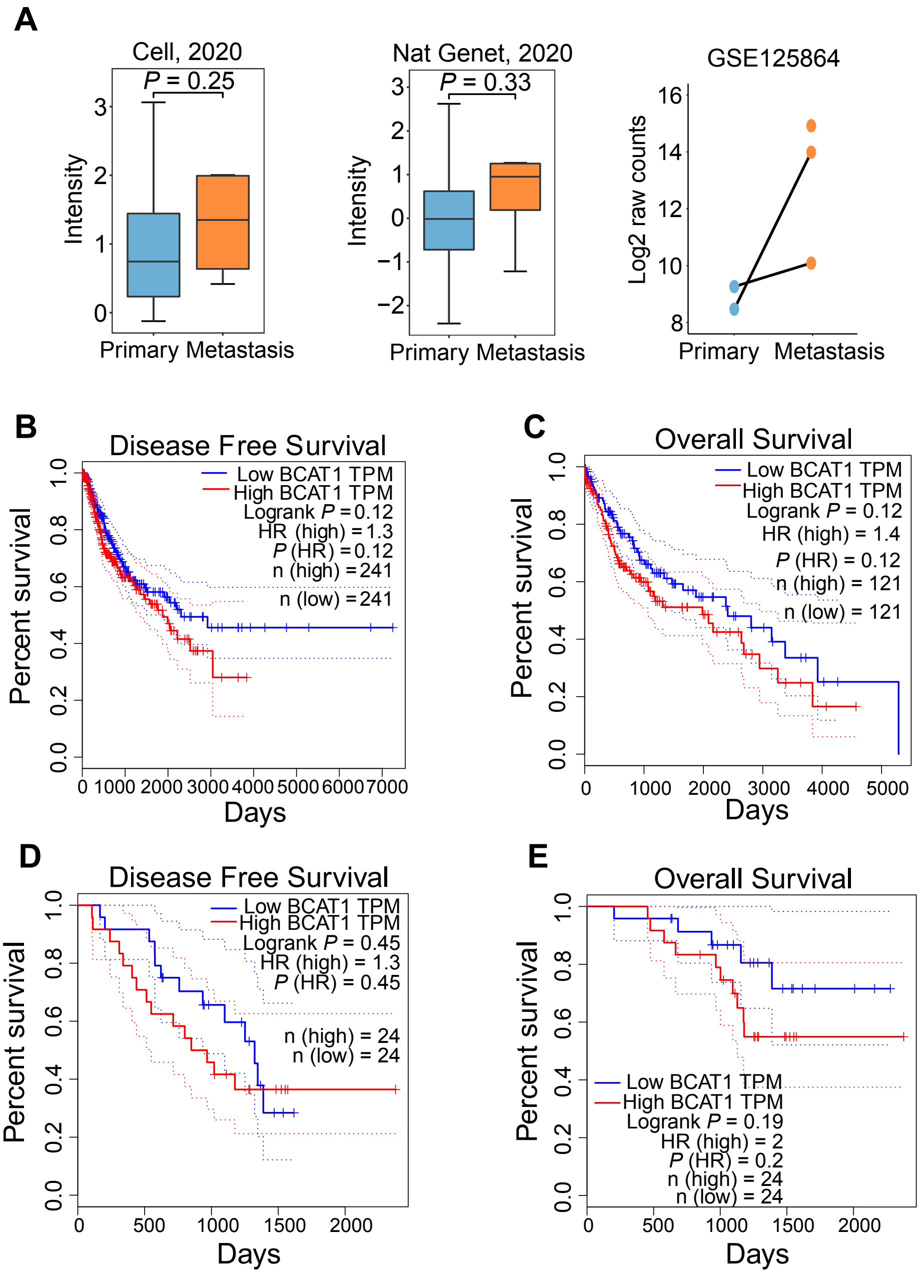

Figure S3. NSCLC patient survival stratified by BCAT1 expression. **(A)** Boxplot and dotplot for BCAT1 expression in 3 datasets [5, 32, 33]. The data from Cell, 2020 was analyzed using Wilcoxon rank sum test since it failed the normally test, and the data from Nat Genet, 2020 was analyzed using Students' *t*-test. **(B)** DFS of LUAD and LUSC patients stratified by 75 quantile expression of BCAT1 from the TCGA datasets. **(C)** OS of LUSC patients stratified by 75 quantile expression of BCAT1. **(D and E)** DFS and OS curves using the dataset from Cell, 2020 [4].

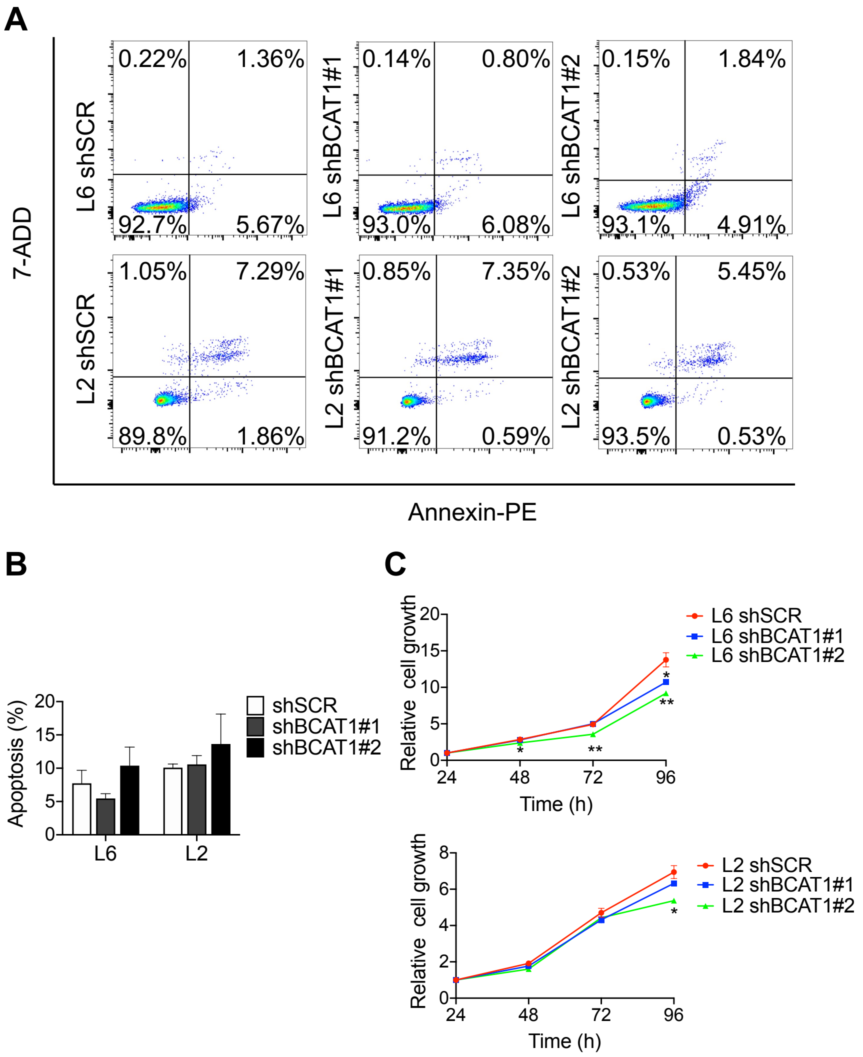

Figure S4. The effect of knocking down BCAT1 on cell proliferation and apoptosis. **(A)** FACS analysis of A549 cells after co-staining with annexin-PE and 7-ADD. Annexin-PE positive and 7-ADD positive or negative cells were counted as apoptotic cells. n = 3. **(B)** Student's *t*-test analysis of data from (A). **(C)** Growth curve of cells expressing either scrambled shRNA or shBCAT1. * $P < 0.05$, n = 3.

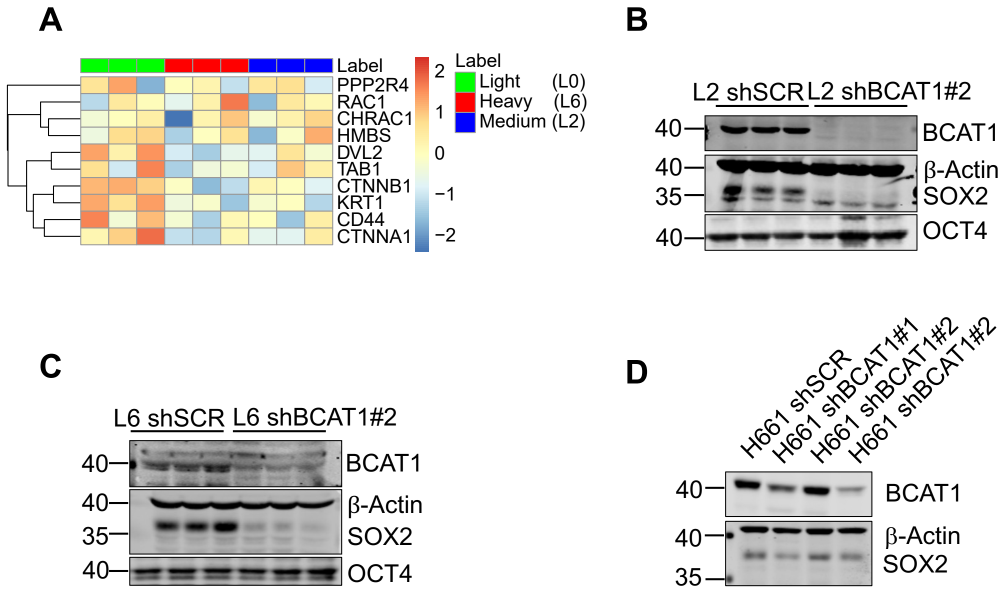

Figure S5. Expression of EMT markers and stemness factors. **(A)** Expression of protein involved in Wnt signaling from SILAC data. **(B, C)** Western blot analysis of SOX2 and OCT4 in L2 (B) and L6 (C) cells after knocking down BCAT1 with the second targeting sequence. **(D)** Western blot of SOX2 in H661 cells expressed sh-BCAT1.

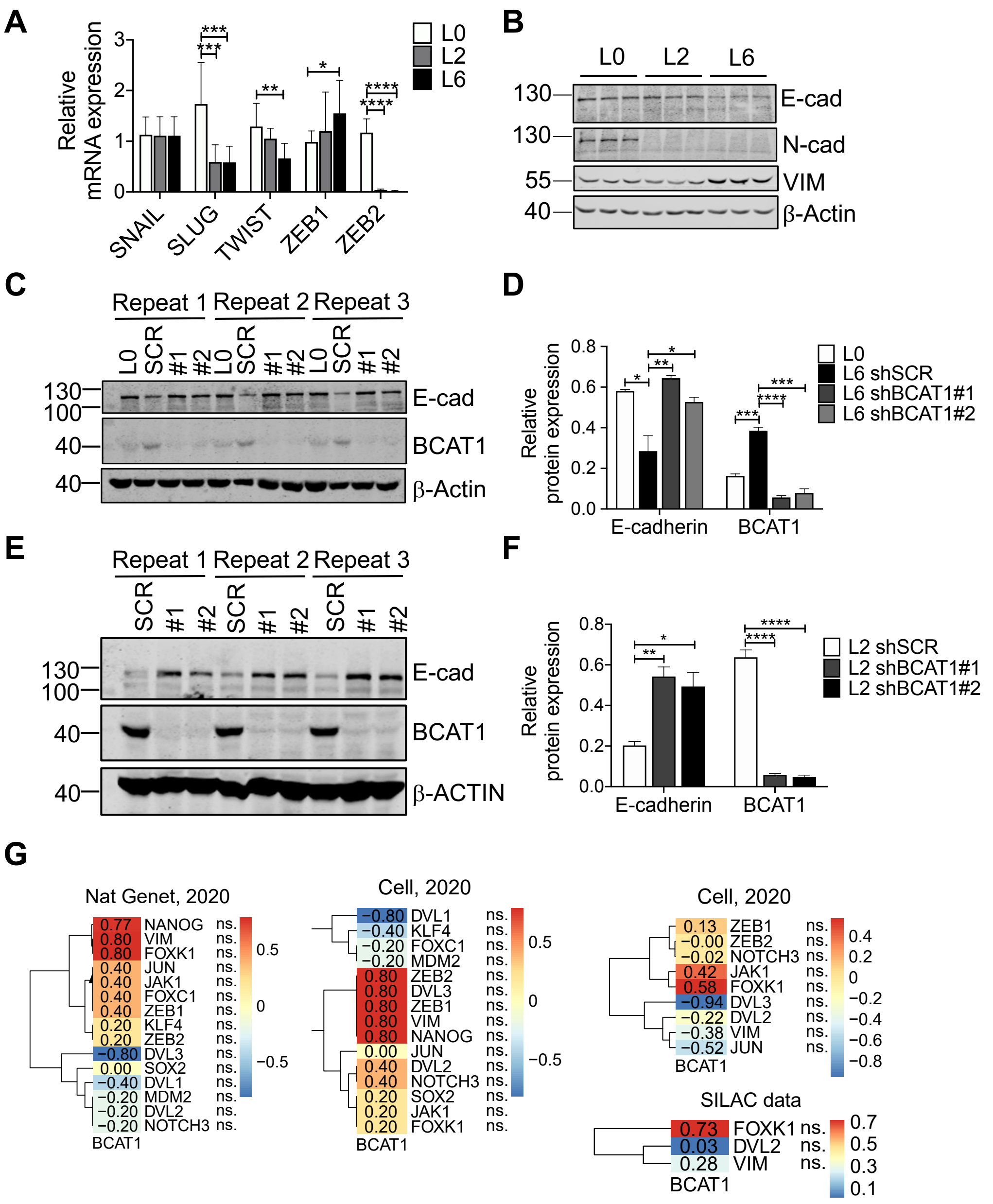

Figure S6. Expression of EMT markers and transfactors. **(A-B)** Relative mRNA expression of EMT related transcription factors (A) and protein markers (B) in A549 cells. **(C-F)** Expression of E-cadherin at the protein level in L2 and L6 cells expressing shBCAT1. **(G)** Correlation analysis between BCAT1 and EMT- or stemness-associated genes from RNA-seq data (left and middle) [32], or proteomic data (right) [5], as well as our SILAC data. Correlation coefficients were shown in the box and *P* values were shown on the side. ns.: none significant. RNA-seq data from Nat Genet, 2020 and Cell, 2020 were analyzed using Spearman's rank correlation, while the other two were analyzed using Pearson correlation.

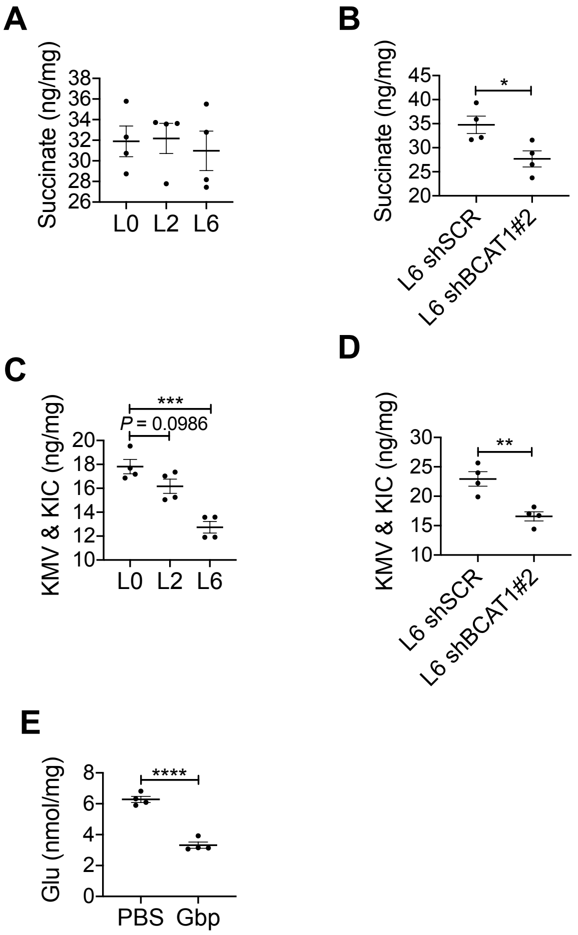

Figure S7. Quantification of succinate and BCKAs. **(A-B)** Cellular succinate levels normalized to total protein among of cells. n=4. **(C-D)** Cellular levels of keto-acid KMV and KIC normalized to total protein among of cells. Since the two isoforms were not separable by either HPLC or by MS, the summed intensities were measured. **(E)** Cellular glutamate concentration treated with or without gabapentin (20 mM, 24 h).

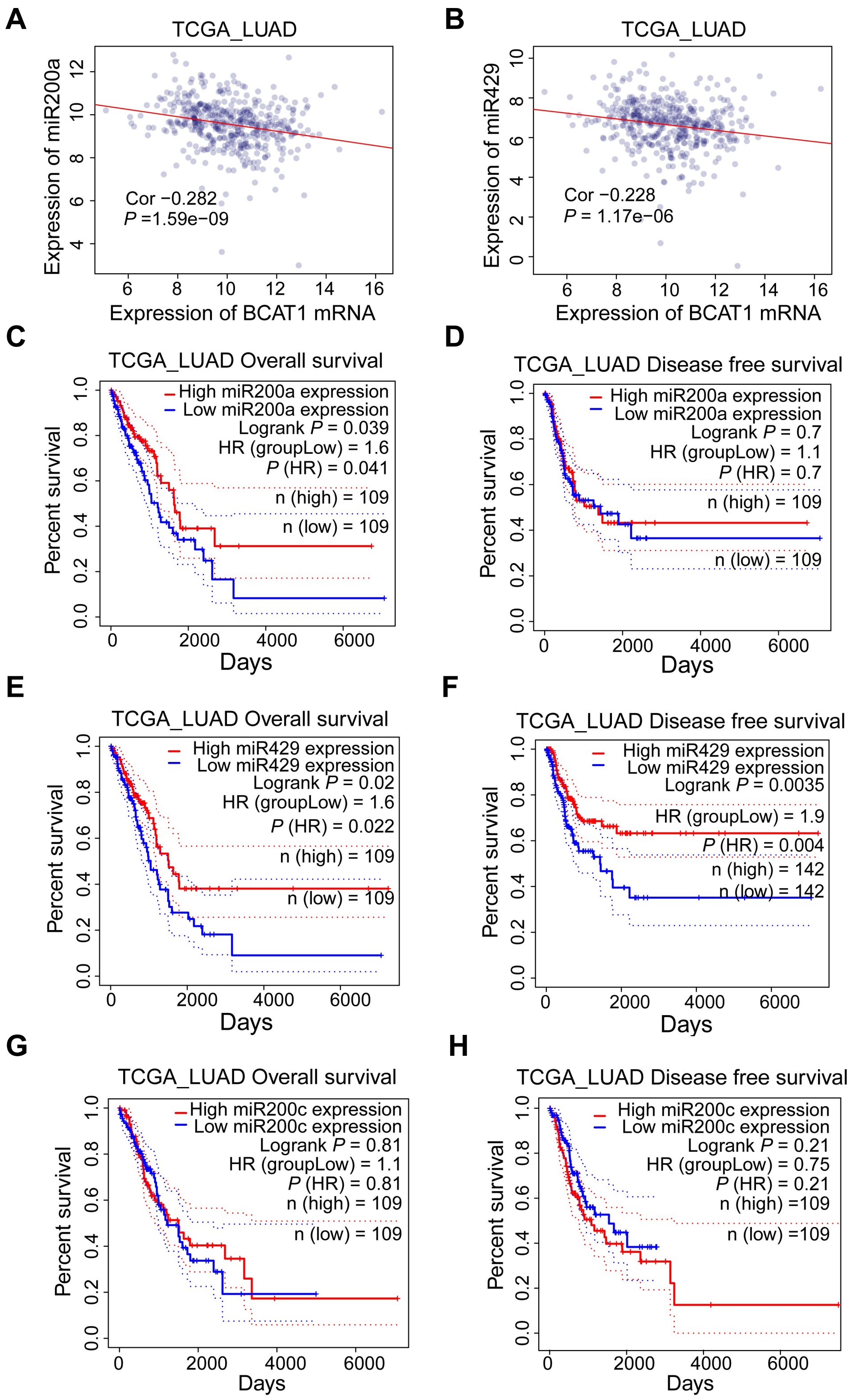

Figure S8. MiR200 family members predict prognosis of NSCLC patients. **(A-B)** Pearson correlation analysis between BCAT1 and miR200 family members miR200a (A) or miR429 (B). **(C-H)** Kaplan-Meier OS or DFS curve of LUAD patients stratified by 75% quantile expression of miR200 family members. The expression data and patient information were downloaded from the TCGA datasets.

Table S1. Primer sequences for all experiments.

Gene	Primer sequence forward 5'-3'	Primer sequence reverse 5'-3'
For QPCR		
BCAT1	GTGGAGTGGTCCTCAGAGTTT	AGCCAGGGTGCAATGACAG
CPS1	AATGAGGTGGGCTTAAAGCAAG	AGTTCCACTCCACAGTTCAGA
PYCARD	TGGATGCTCTGTACGGGAAG	CCAGGCTGGTGTGAAACTGAA
ASS1	TCCGTGGTTCTGGCCTACA	GGCTTCCTCGAAGTCTTCCTT
TAGLN	AGTGCAGTCCAAAATCGAGAAG	CTTGCTCAGAATCACGCCAT
SOX2	CGAGTGGAAACTTTTGTCGGA	TGTGCAGCGCTCGCAG
OCT4	ACCGAGTGAGAGGCAACC	TGAGAAAGGAGACCCAGCAG
NANOG	CAACCAGACCCAGAACATCC	TTCCAAAGCAGCCTCCAAG
SNAIL	CTTCCAGCAGCCCTACGAC	CTGAGGATCTCTGGTTGTGGT
SLUG	AGATGCATATTCGGACCCAC	CCTCATGTTTGTGCAGGAGA
TWIST	GCCAGGTACATCGACTTCCTCT	TCCATCCTCCAGACCGAGAAGG
ZEB1	GATGATGAATGCGAGTCAGATGC	ACAGCAGTGTCTTGTTGTTGT
ZEB2	CAAGAGGCGCAAACAAGCC	GGTTGGCAATACCGTCATCC
miR200c-3p	ACACTCCAGCTGGG UAAUACUGCCGGGUAAUGA	CTCAACTG GTGTCGTGGA
miR-429	ACACTCCAGCTGGG TAATACTGTCTGGTAAAA	CTCAACTG GTGTCGTGGA
miR21-5p	ACACTCCAGCTGGGTAGCTTAT CAGACTGATG	CTCAACTG GTGTCGTGGA
U6	CTCGCTTCGGCAGCACA	AACGCTTCACGAATTTGCGT
For reverse transcription (Stem-loop)		
miR-200c-3p	CTCAACTGGTGTCGTGGAGTCGGCAATTCAGTTGAG TCCATCAT	
miR-429	CTCAACTGGTGTCGTGGAGTCGGCAATTCAGTTGAG ACGGTTTT	
miR21-5p	CTCAACTGGTGTCGTGGAGTCGGCAATTCAGTTGAG TCAACATC	
U6	AACGCTTCACGAATTTGCGT	

For BCAT1 knock down		
shBCAT1#1	CCGGCCCAATGTGAAGCAGTAGATACTC GAGTATCTACTGCTTCACATTGGGTTTTT	AATTAAAAACCCAATGTGAAGCAGTAGA TACTCGAGTATCTACTGCTTCACATTGGG
shBCAT1#2	CCGGCCTGTGTTGTTTGCCCAGTTTCTC GAGAAACTGGGCAAACAACACAGGTTTTT	AATTAAAAACCTGTGTTGTTTGCCCAGT TTCTCGAGAAACTGGGCAAACAACACAGG

Table S4. Clinical information for samples analyzed by TMT-based proteomics

Patient No.	Age	Sex	Metastatic site	Primary site	Histological type	Therapy
FFPE sections						
No.1	65	M	Chest wall	Superior lobe of left lung	Lung squamous carcinoma	Radiotherapy
No.2	53	M	Lymph node of right cervical	Superior lobe of left lung	Neuroendocrine neoplasm	Postoperative docetaxel and carboplatin chemotherapy
No.3	67	F	Lymph node of left cervical	Inferior lobe of left lung	Lung adenocarcinoma	Surgical operation
No.4	65	M	Chest wall	Inferior lobe of left lung	Lung squamous carcinoma	Docetaxel and carboplatin chemotherapy
Frozen tissues						
No.5	62	F	None	Superior lobe of right lung	Papillary infiltrating adenocarcinoma	Surgical operation
No.6	61	F	lymph node	Superior lobe of right lung	Papillary infiltrating adenocarcinoma, with acinar type, micropapillary type	Surgical operation
